
JOURNAL INFORMATION
==============================
NLM Title Abbreviation: Atlanta Med Surg J
NLM Journal ID: 0001010

Atlanta Medical and Surgical Journal

Publisher: Biomedical Journal Digitization Project

ARTICLE INFORMATION
==============================
PMCID: PMC8823834
PMID: 35823953
Article version: 1
Subjects: Editorial and Miscellaneous

Paris Faculty of Medicine

Print publication date: 1867 Jan
Volume: 7B
Volume Series: New Ser
Issue: 11
First page: 528
Last page: 528
License: This work is free of known copyright restrictions. For more information, please see PMC Back Issue Digitization.
License URL: https://creativecommons.org/publicdomain/mark/1.0/

==============================

*Paris Faculty of Medicine.*—This body, it is said, is likely soon to undergo considerable change in its *personnel*, and not before this is required, if it be true, as stated, that no less than seven professors are incapacitated by age or illness, not to mention M. Trosseau's imminent resignation. The chair of clinical surgery lately held by M. Jobert (de Lambelle), now hopelessly insane, is to be immediately filled.—*Ibid.*

---

### MASSEY & HERTY,

### Whitehall Street,

### ATLANTA, GEORGIA,

WHOLESALE AND RETAIL DEALERS IN

### Pure Drugs, Chemicals, Patent Medicines,

Perfumeries, Toilet Articles, Stationery, Cigars

**Wines, Liquors, Dye-Stuffs, Paints,**

OILS, BRUSHES, GLASS, PUTTY, &C.

Coal Oil and Lamps, Machine and Tanners' Oil always on hand.

☞ *Will duplicate all Orders made South.*
